# Molecular Interplay between Dormant Bone Marrow-Resident Cells (BMRCs) and CTCs in Breast Cancer

**DOI:** 10.3390/cancers12061626

**Published:** 2020-06-19

**Authors:** Debasish Boral, Haowen N. Liu, S. Ray Kenney, Dario Marchetti

**Affiliations:** 1 Center for Immunotherapy Research, Houston Methodist Research Institute, Houston, TX 77030, USA; dboral@houstonmethodist.org; 2 Biochemistry lab, Complete Genomics Inc., San Jose, CA 95134, USA; hliu@completegenomics.com; 3 Division of Molecular Medicine, Department of Internal Medicine, The University of New Mexico, Health Sciences Center, Albuquerque, NM 87131, USA; SRKenney@salud.unm.edu

**Keywords:** Bone Marrow-Resident Breast Cancer Cells (BMRCs), Circulating Tumor Cells (CTCs), bone marrow (BM), CTC-derived xenograft (CDX), mTOR pathway, mTORC1/mTORC2 signaling, RICTOR, CTC-associated dormancy

## Abstract

Despite widespread knowledge that bone marrow-resident breast cancer cells (BMRCs) affect tumor progression, signaling mechanisms of BMRCs implicated in maintaining long-term dormancy have not been characterized. To overcome these hurdles, we developed a new experimental model of clinical dormancy employing patient-isolated Circulating Tumor Cells (de novo CTCs) and their injection in xenografts with subsequent tumor monitoring and CTC characterization (ex vivo CTCs). We hypothesized that significant distinctions exist between signaling pathways of bone marrow-homing vs metastasis-competent CTCs upon transplantation in xenografts. Comparative transcriptomic analyses of ex vivo vs de novo CTCs identified increased mTOR signaling—a critical pathway frequently dysregulated in breast cancer and implicated in cell survival and dormancy—with contrasting actions by its two complementary arms (mTORC2/mTORC1). Heightened mTORC2 downstream targets augmented quiescent CTCs (Ki67−/RBL2+ cells) in paired breast cancer tissues, along with high mTORC2 activity in solitary BMRCs and tissue-resident CTCs. Further, shRNA mediated the knockdown of RICTOR, an essential component of mTORC2, and augmented Ki67/PCNA biomarker expression and proliferation. Collectively, these findings suggest that the balance between mTORC1 vs mTORC2 signaling regulates CTC-associated mitotic and/or dormancy characteristics.

## 1. Introduction

**Figure 1:**
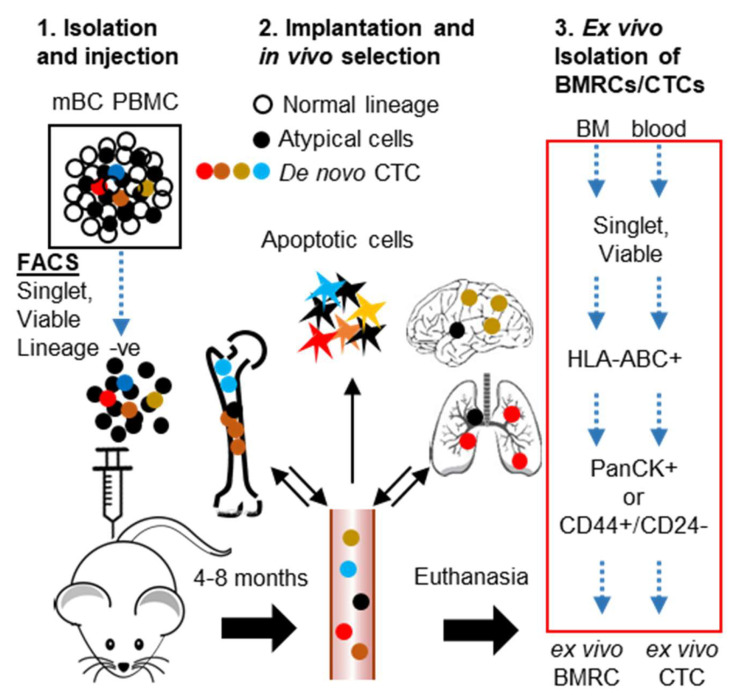
Strategy for in vivo enrichment of mBC patient-derived CTCs and isolation of ex vivo BMRCs and CTCs. This strategy employs: 1. Doublet and dead cell removal, followed by lineage depletion of patient peripheral blood mononuclear cells and injection of de novo CTC-enriched cells into NSG mice; 2. In vivo selection of CTCs (ex vivo CTCs) following their dissemination to bone marrow (BMRCs) and to target organs. A portion of CTCs capable of BM/target organ homing is expected to be shed back into circulation; 3. Collection of tissues and the isolation of ex vivo BMRCs and CTCs by FACS. The strategy for biomarkers and FACS selection steps of ex vivo BMRCs and CTCs is highlighted in the red box.

Patients with metastatic breast cancer (mBC) have a 5-year survival rate of only 28% compared to 99% in patients with non-metastatic cancer [1]. The pathogenesis of metastatic dissemination starts early in the carcinogenic process, in a step referred to as ‘metastatic seeding’, when BC cells invade through the basement membrane and intravasate into blood as circulating tumor cells (CTCs) [2,3,4,5]. While the overwhelming majority of these CTCs die in the circulation, some CTCs are able to survive. These CTCs persist in distant organs as clinically undetectable micro-metastases, either as quiescent (G_0_) single cells or as dormant foci where the total number of cells undergoing proliferation is balanced by cells undergoing apoptosis [6,7,8]. Multi-organ metastatic seeding can be a synchronous event in which all target organs are seeded simultaneously [9,10]. However, it is likely that at least a sub-population of BC cells home to, and reside in, the bone marrow (BM) where they accumulate genomic instability over time, gradually acquiring the ability to colonize additional organs [11,12,13]. Despite this knowledge, properties and mechanisms implicated in the maintenance of patient-derived BM-resident BC cells (BMRCs) remain under-investigated. Although solitary breast cancer cells are known to persist in BM and visceral organs after resection of the primary tumor and systemic chemo/radiotherapy, tissue biopsies or BM aspiration to capture solitary mBC cells are not readily feasible. Further, despite several reports of ex-vivo CTC culture, there is no model of clinical dormancy that can comprehensively emulate the entire spectrum of heterogeneous CTC clones present in mBC patients [14,15,16]. Further, because cell line-based studies do not reflect the mechanisms involved in maintenance of prolonged dormancy due to accumulated genomic instability, and small animal studies do not replicate the human response to micro-metastases from invaded tissues and immune surveillance. The duration of clinical remission shows marked inter-patient variability, making it is virtually impossible to design and implement a universal screening tool for identifying metastatic progression before secondary sites become symptomatic. Therefore, to make substantial improvements in recurrence-free survival of BC patients, it is critical to identify the CTCs that can survive and act as seeds of metastatic recurrence before the onset of symptomatic metastasis. Here we report a three-tier strategy for (1) the unbiased derivation of CTC-enriched populations from the blood of mBC patients, the injection of these populations in experimental mouse models (2), and the in vivo expansion and selection of tissue-resident CTCs, which allow for (3) the ex vivo isolation and characterization of BMRCs and CTCs (Figure 1). We report the development of streamlined workflows to dissect the molecular pathways implicated in BC CTC-associated dormancy at single-cell level, as well as the determination of initial CTC colonization capabilities in vivo.

## 2. Results

### 2.1. Isolated Patient CTCs Are Characterized by Proliferative/Epithelial Markers or Quiescent/Stem-Like Markers

**Figure 2:**
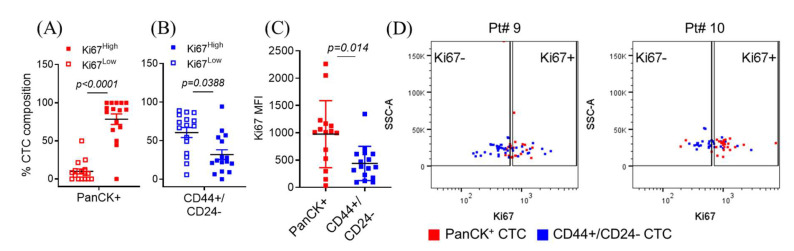

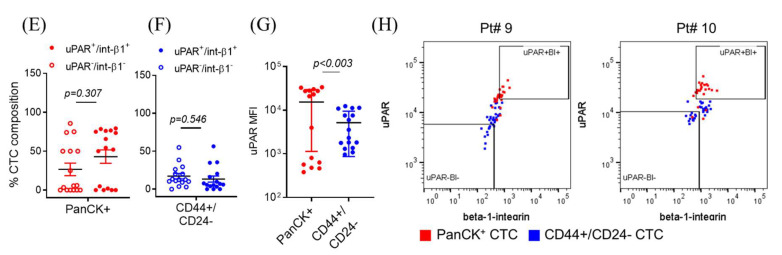
Stem-like (CD44+/CD24−) breast cancer CTCs are more likely to be mitotically inactive (Ki67−) than epithelial (PanCK+) CTCs. (**A**) Distribution of Ki67 expression in epithelial and (**B**) stem-like CTCs across 16 patient samples. (**C**) Median fluorescence intensity (MFI) of Ki67 in CTCs. (**D**) Expression of Ki67 in epithelial and stem-like CTCs. Representative dot plots from patient #9 and #10 shown. (**E**) Combinatorial expression of uPAR and int-β1 in epithelial and (**F**) stem-like CTCs. (**G**) MFI of uPAR in CTCs. (**H**) Combinatorial expression of uPAR and int-β1in epithelial and stem-like CTCs. Representative dot plots from patient #9 and #10 are shown. Dot plots from all 16 patients are shown in Figure S1. CTCs from 16 metastatic breast cancer patients were used for these analyses. *P*-values were calculated using paired-t tests.

CellSearch^TM^, the only FDA-cleared platform for clinical CTC testing, captures breast cancer CTCs based on the combination of EpCAM+ and cytokeratin+ (epithelial origin), and CD45− (for leukocyte depletion) biomarkers, leading to exclusion of non-epithelial or stem-like CTCs [17]. CellCollector^®^, the CE and CFDA-cleared platform, also isolates CTCs based upon EpCAM expression [18]. Additionally, there are multiple technologies in various stages of development to isolate and interrogate disparate CTCs [19]. We consider the use of multi-parametric flow cytometry a rigorous and reproducible approach [14,20] that allows evaluation and capture of the entire spectrum of CTCs and/or abnormal mononuclear cells. We have previously reported that a FACS-based workflow employing the depletion of multiple normal cell lineages (CD45, CD73, CD34, CD105, CD90), along with the positive selection for both epithelial (PanCK+), as well as stem-like (CD44+/CD24−) CTCs, capturing 2–8 fold higher number of stem-like CTCs than epithelial CTCs [21]. Multiple studies have demonstrated that the presence of epithelial CTCs correlate with worse prognosis of metastatic breast cancer, but the role/s of stem-like CTCs remain relatively unknown. Therefore, flow cytometric analyses were performed to determine whether a correlation exists between CTC surface phenotypic markers (epithelial vs stem-like) and the underlying mitotic state in 16 mBC patients. We found that ~83% of epithelial (PanCK+) CTCs were Ki67^High^ while only 32% of stem-like (CD44+/CD24−) CTCs were Ki-67^High^ (Figure 2A–D, and Figure S1). Interestingly, 88% of CTCs were of the stem-like phenotype (Figure S2A,B). Of this, 61% (53% of the total CTC population) were Ki67^Low^ (Figure 2B). We also checked the combinatorial expression patterns of uPAR and integrin-β1 (int-β1), two established biomarkers of CTC dormancy and metastatic competence [21,22,23]. When measured individually, uPAR expression was significantly higher in epithelial CTCs (Figure 2G), while int-β1 expression was similar between epithelial vs stem-like CTCs (Figure S2C). However, upon combining the two biomarkers to identify uPAR+/int-β1+ vs uPAR−/int-β1− CTC groups (Figure 2H and Figure S1), we did not find a significant difference in the proportion of stem-like and epithelial CTCs in these two groups (Figure 2E,F). Combined, these data indicate that the majority of CTCs found in metastatic breast cancer patients maintain a stem-like (CD44+/CD24−) state, while staying mitotically inactive; whereas a smaller sub-population of CTCs are of an epithelial (PanCK+) phenotype, with a higher proliferative index. We built upon these data to determine what molecular pathways may be involved in the switch between quiescence and proliferation, and examined whether the cellular environment plays a role in determining the proliferative status.

### 2.2. Isolation and Characterization of Ex Vivo BMRCs and CTCs

**Figure 3:**
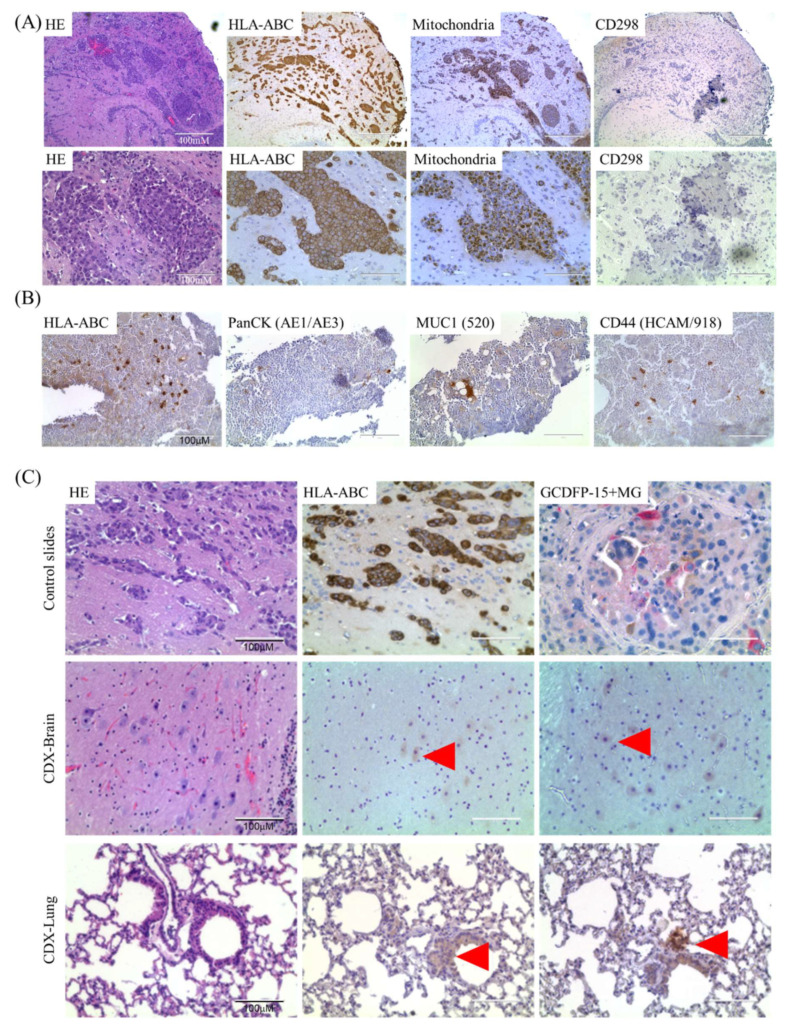
HLA-ABC identifies cells of human origin in xenografted mice. (**A**) Serial sections of brain macro-metastasis obtained 6 weeks after intracardiac injection of 1 × 10^5^ MDA-MB-231BR cells. Left to right: Hematoxylin-eosin (HE) staining, HLA-ABC, anti-mitochondrial antibody, CD298 staining. Upper panels show lower magnification, and lower panels show higher magnification imaged. Note that both HLA-ABC and the anti-mitochondrial antibody identify the same malignant lesions identified in the HE stained section. CD298 did not identify the metastatic masses. Also note the localization of HLA-ABC staining in the cell membrane while the anti-mitochondrial antibody localized to the cytoplasm. (**B**) Serial sections of mouse bone marrow 3 weeks after intra-cardiac injection of 1 × 10^5^ MCF10A cells. Left to right: HLA-ABC, PanCK, Mucin1, CD44 IHC. Note HLA-ABC identifies both PanCK+ as well as CD44+ cells. (**C**) CTC-derived xenograft (CDX) brain and lung tissues show solitary CTC-derived cells as micro-metastatic seeding. Top panels show staining patterns in positive control slides. Left: HE, center: HLA-ABC, right: GCDFP-15+MG. MDA-MB-231BR-derived brain metastatic lesion used a positive control for HE and HLA-ABC staining. HER2+ PDX mouse brain tissue was used as positive control for GCDFP15/MG staining. GCDFP-15+MG shows brown (HRP-DAB) staining for mouse antibody clones 23A3 (anti-GCDFP-15) and 304-1A5 (anti-MG), and red (AP-Red) staining for rabbit clone 31A5 (anti-MG). Middle and bottom panels show serial sections of CDX-Brain and CDX-Lung tissues stained for HE, HLA-ABC, or GCDFP-15+MG IHC, respectively. Red arrowheads indicate positive staining using either HLA-ABC or GCDFP-15+MG, showing solitary mBC CTC-derived cells in brain and lung micro-metastases (scale bars = 100 μm).

Metastatic seeding is an early event initiated when cancer cells that are shed from the primary tumor, enter into the blood stream as CTCs, and travel to distant organs where they can remain dormant as disseminated tumor cells for indefinite lengths of time [24,25,26,27]. We anticipated that CTCs with colonization potential will home to BM and/or visceral organs and survive or proliferate, whereas CTCs without colonizing potential (along with other non-malignant cells) will become apoptotic. Since the bone marrow serves as the foremost reservoir for these CTCs, we developed a preclinical model employing a three-tiered enrichment strategy (FACS - in vivo selection for 4–8 months - FACS) for isolating ex vivo BMRCs using mBC patient-derived CTCs (Figure 1). As a pre-requisite for this model, we first developed a method to identify and isolate cells of human origin from the bone marrow of xenografted mice. To do so, we injected mice with 1 × 10^5^ MDA-MB-231BR or MCF10A mammary epithelial cell lines via the intracardiac route, euthanized them at regular intervals, and checked for the presence of metastatic cells by IHC. We evaluated HLA-ABC, anti-mitochondrial antibody, and CD298 (Figure 3A). We found that HLA-ABC identifies human cancer cells in xenografted mice, and localizes in the cell membrane, making it ideal for use with flow cytometry. IHC examination of xenografted mouse BM showed that HLA-ABC identified solitary as well as scattered clusters of MCF10A cells within the mouse bone marrow (Figure 3B). As expected, the majority of MCF10A cells stained positive for CD44 (primate-specific anti-CD44 antibody), while solitary cells also stained positive with PanCK (Figure 3B). The persistence of MCF10A cells within the BM of xenografted mice demonstrates that transformed but non-tumorigenic mammary epithelial cells can survive within the BM microenvironment.

**Figure 4:**
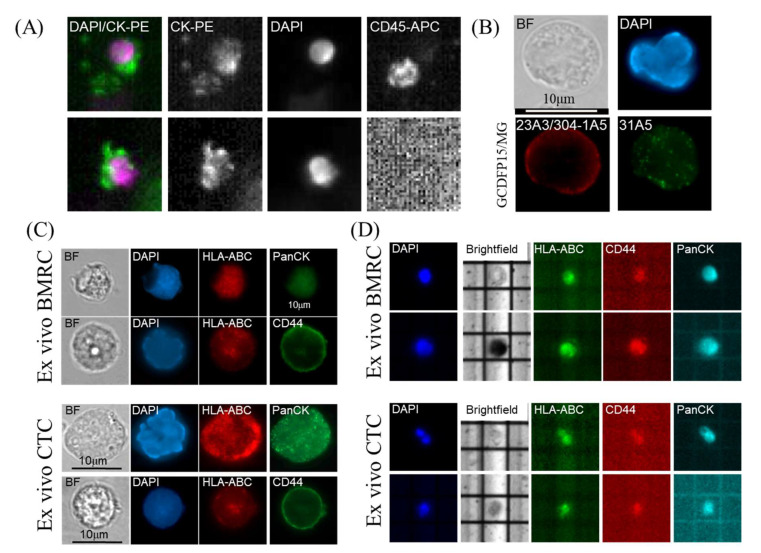
Validation of ex vivo BMRC/CTC using complementary imaging platforms. (**A**) Detection of ex vivo human CTCs by CellSearch CTC (epithelial) kit. The kit enumerates CTCs using DAPI, Pan-cytokeratin-PE (CK-PE), and CD45-APC staining. Upper panel shows a CTC clustering with a CD45+ cell (lymphocyte); lower panels show an individual CTC. Image resolution achieved in accordance with the USFDA-approved CellSearch platform. (**B**) Immunofluorescent (IF) staining of ex vivo BMRC using GCDFP15/MG cocktail. Image of a BMRC showing red IF staining against mouse primary antibody clones 23A3 (GCDFP-15) and 304-1A5 (MG), and green IF staining for rabbit clone 31A5 (MG). (**C**) IF images of single ex vivo epithelial and stem-like BMRCs and CTCs. (**D**) DEPArray images of ex vivo epithelial and stem-like BMRCs and CTCs. Image magnification: 10×. The DEPArray platform allows the capture, visualization, and downstream interrogation of single CTCs. Since the image resolution and magnification obtained from FDA-cleared CellSearch and DEPArray platforms cannot be altered by the user, we used IF to provide a high-resolution image of captured ex vivo BMRC/CTCs.

Having validated that HLA-ABC can identify both PanCK+ and CD44+ breast cancer cells in mouse bone marrow, we depleted doublets (SSC-H v SSC-A) and dead cells (DAPI+), normal blood cell lineages (leukocytes: CD45+, lymphocytes, mesenchymal, endothelial progenitors: CD34+, macrophages, fibroblasts CD90+/CD105+, mesenchymal, hematopoietic stem cells, NK cells: CD73+) by FACS, and implanted the residual CTC-enriched/lineage-negative cells into NOD scid-gamma (NSG) mice by intracardiac injection [20,28,29]. Mice were euthanized after 4–8 months of in vivo selection, and target organs (brain, lung, liver, spleen) were evaluated for the presence of tumor cells by H/E staining and IHC using HLA-ABC (to prove human origin) and GCDFP-15/Mammaglobin (MG) cocktail (to prove mammary origin). Blood and BM were subjected to FACS to isolate ex vivo BMRCs/CTCs (Figure 1). Though we did not find overt macro-metastases, upon careful scrutiny, we detected solitary BC cells in brain tissue and micro-metastatic foci in brain (Figure 3C middle panel) and lung (Figure 3C, bottom panel). CTCs were also identified in blood of CTC-derived xenograft (CDX) mice by the CellSearch platform (Figure 4A). Additionally, immunofluorescent (IF) staining using the GCDFP15/MG cocktail confirmed that ex vivo BMRCs were of mammary origin (Figure 4B). Thus, we implemented this strategy on six mBC patients (two each of ER+/PR+, HER2+, and triple-negative BC), and isolated ex vivo BMRCs and CTCs from mice (patient parameters and BMRC/CTC numbers are provided in Table 1). Interestingly, a portion of putative ex vivo BMRCs and CTCs expressed both PanCK and CD44 on their cell surface, suggesting an epithelial-to-mesenchymal transition (EMT) state captured by both biomarkers, which is consistent with previously reported data [30,31]. The identity of isolated cells was validated by immunocytochemistry (ICC) (Figure 4C) and by the automated, antigen-agnostic, rare-cell DEPArray^TM^ platform (Figure 4D). In line with the flow cytometry analyses, a significant pool of putative ex vivo CTCs and BMRCs expressed both PanCK as well as CD44 on their cell surface.

### 2.3. mTOR Signaling Is Upregulated in Ex Vivo CTCs

**Figure 5:**
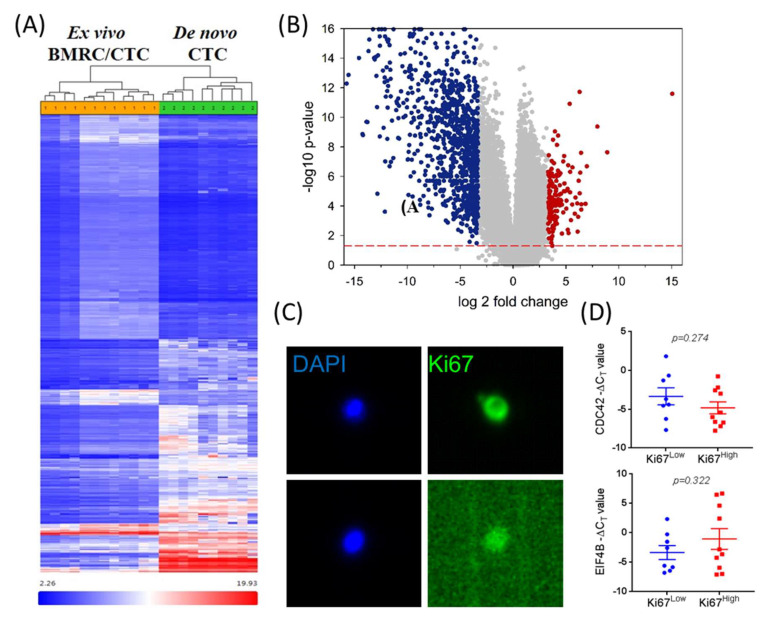
The comprehensive transcriptomic analysis of ex vivo BMRC/CTC vs de Novo CTC populations. (**A**) Heat map showing differential gene expression in ex vivo BMRC/CTC populations vs de novo CTCs reveal 4,309 differentially regulated genes, as analyzed by one-way ANOVA (fold change −4< > 4, *p*-value < 0.05). (**B**) Volcano plot showing significantly upregulated vs downregulated genes. Microarray data is available from the Gene Expression Omnibus as GSE150624. (**C**) Representative images of ex vivo CTCs isolated and interrogated in parallel for Ki67 expression by the DEPArray. Upper panel Ki67^High^ CTC, lower panel: Ki67^Low^ CTC. Image magnification: 10×. Note that DEPArray positive/negative IF values are determined by CellBrowser^TM^ software, which calculates multiple parameters (background, mean fluorescence, etc.) during cell detection. Intensity threshold settings are then automatically applied to captured images, equalizing fluorescence levels in all images, but without altering primary fluorescence intensity values. (**D**) DEPArray-isolated CTCs were subjected to qPCR analysis for mTORC gene expression targets: CDC42 (top) and E1F4B (bottom) (CDC42 and EIF4b are targets of mTORC2 and mTORC1 respectively) [23,24]. Ki67^Low^ (*N* = 8) CTCs had increased CDC42 expression (*p* = 0.274) while Ki67^High^ (*N* = 10) CTCs had augmented EIF4B expression (*p* = 0.322).

Because we previously characterized the transcriptomic signature of de novo CTCs (DAPI-, lineage-, PanCK+ or CD44+/CD24− cells) [21], we maintained the same biomarker definition, FACS selection, and microarray (Affymetrix HTA_2.0 platform) to isolate ex vivo BMRCs/CTCs (Figure 1, red box), and performed whole genome transcriptomic microarrays. We considered a three-way comparison between transcriptomes of de novo CTCs (GSE99394) ex vivo BMRCs, and ex vivo CTCs to reflect differences in signaling mechanisms acquired during the bone-homing vs. organ colonization and selection in vivo. Transcriptomic analysis revealed that the gene expression signatures of ex vivo BMRCs and ex vivo CTCs were largely similar. Conversely, we found 279 genes which were differentially regulated but clustered together by comparing ex vivo BMRCs/CTCs to de novo CTCs (Figure 5A,B). Gene expression levels of de novo vs ex vivo cells were subjected to pathway enrichment analysis. This identified a decrease in signaling mechanisms related to cell proliferation, invasion and metastasis, along with an overall decrease in protein translation, all features which are suggestive of a dormant status, as provided in Table 2. We identified mTOR signaling, a critical pathway frequently dysregulated in breast cancer and implicated in cell survival and dormancy through its two complementary arms (mTORC1 and mTORC2), as the most significantly activated signaling pathway of ex vivo cells. Furthermore, two out of the five most significant upstream regulators of ex vivo cells were rapamycin and RICTOR (Table 3). Because rapamycin is known to inhibit mTORC1 without affecting mTORC2, and RICTOR is the essential subunit of mTORC2 complex, the augmented mTOR signaling (Table 2) is mTORC2 [32,33,34,35,36].

### 2.4. Differential mTORC Activities in Quiescence vs Proliferation Properties of CTCs and Metastatic Tumors

Next, we investigated whether differential mTORC activities could be related to quiescence vs proliferation properties employing either CTCs or primary/metastatic BC tumors. Despite sharing some common targets, mTORC1 and mTORC2 have a distinct set of target genes, e.g., EIF4B (mTORC1) and CDC42 (mTORC2). We isolated putative CTCs from blood employing FACS followed by IF staining and DEPArray, as previously reported by our laboratory [21]. This included the simultaneous selection for CTC cell surface (CD44), cytoplasmic, (CK), and nuclear (DAPI and Ki67) biomarkers using single-step flow cytometry followed by immunofluorescence analysis. Of note, the isolation of single Ki67^Low^ and Ki67^High^ CTCs was achieved by a sequential FACS workflow followed by ethanol fixation, nuclear staining and DEPArray [21]. This resulted in the isolation of single putative Ki67^Low^ and Ki67^High^ CTCs (Figure 5C). Whole transcriptomic amplification of these cells, followed by qPCR analysis of mTORC1 and mTORC2 target genes, (CDC42 and EIF4B, respectively), was performed (Figure 5D). We detected a trend between Ki67^low^ CTCs and high CDC42 gene expression. Conversely, high EIF4B gene expression levels were found in Ki67^high^ CTCs.

**Figure 6:**
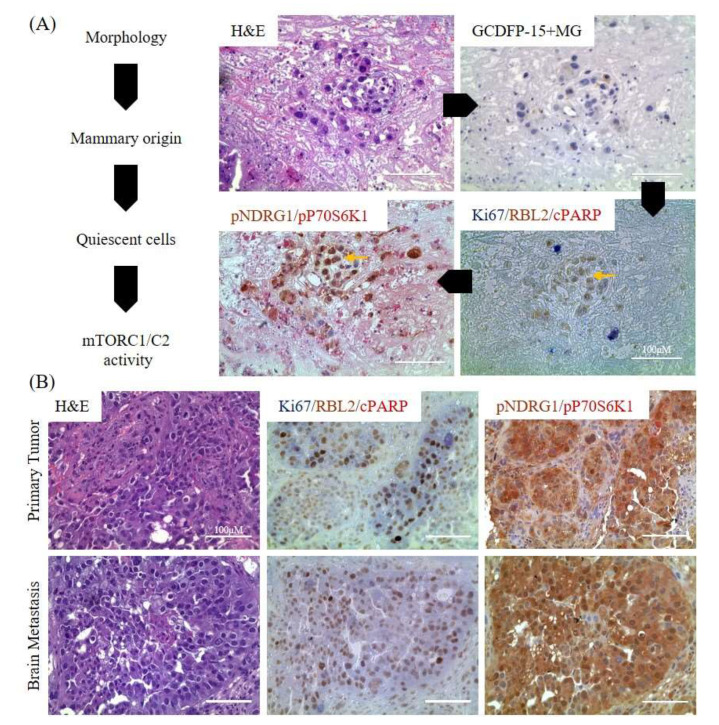

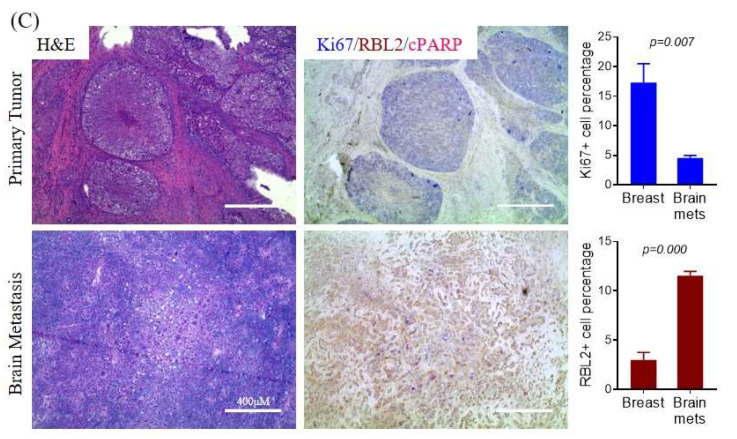
IHC analyses of mTORC2 activity in BC patient samples. Paired primary and metastatic patient BC samples show differences in proliferation and mTORC status. (**A**) Flowchart for IHC evaluation of tumor (left) and images of representative BC brain metastasis serial sections (right) stained for H&E; GCDFP-15+MG (blue) confirming mammary origin of tissue; Ki67/RBL2/cleaved PARP-Asp214 (cPARP), Ki67 (blue, proliferation marker), RBL2 (brown, quiescence marker) [34], cPARP (red, apoptosis marker); and pNDRG1 (brown, mTORC2 status marker), p70S6K (red, mTORC1 status marker) (scale bar = 100 μm). Yellow arrows indicate quiescent cells (RBL2+ and pNDRG1− cells). (**B**) Serial section IHC images of primary and paired brain metastasis for H&E staining, Ki67/RBL2/cPARP for mitotic status, and pNDRG1/pP70S6K for mTORC activity, respectively (scale bar = 100 μm). (**C**) Serial section IHC images of primary and paired brain metastasis for H&E staining; Ki67/RBL2/cPARP to determine cell proliferative, quiescent, and apoptotic status, respectively (scale bar = 400 μm). Primary BC tumors exhibited a significantly greater number of cells expressing Ki67 proliferative marker compared to paired brain metastasis (Ki67+ cells). Conversely, BC brain metastatic tissues exhibited a significantly greater number of cells expressing RBL2 quiescence marker (RBL2+ cells) (*N* = 10; *p* = 0.0007 and *p* < 0.0007, respectively). There was no significant difference in number of cells expressing cPARP apoptosis marker (cPARP+ cells) using the same number of serial sections for IHC.

Additionally, mTORC1 and mTORC2 have a distinct set of downstream phosphorylation substrates—mTORC1 phosphorylates the Thr389 residue on P70S6K, while mTORC2 phosphorylates the Thr346 residue of NDRG1 [37,38,39,40,41]. Accordingly, we investigated specific mTORC1/mTORC2 kinase activities by evaluating their respective phosphoprotein expression levels by IHC analyses using paired primary and brain metastatic BC tissues (Figure 6A). The mitotic status of single BC cells was defined by triple IHC staining using rat anti-Ki67 (proliferation marker), rabbit anti-RBL2/p130 (quiescence marker), and mouse anti-cleaved PARP-Asp214 (apoptosis marker) (Figure 6A) [42]. This permitted the isolation of solitary quiescent (Ki67−/RBL2+/cleaved PARP−) BC cells within tumor masses. Further, we identified quiescent BC cells with mTORC1 vs mTORC2 activity by visualizing respective mTORC1/mTORC2 biomarkers by dual IHC staining using rabbit anti-pNDRG1 and mouse anti-pP70S6K antibodies. We found a significant number of cells which were non-proliferative, non-apoptotic, quiescent cells in serial sections of either primary or matched metastatic BC tumors (Figure 6B). Greater staining for pNDRG1, rather than pP70S6K, suggests increased mTORC2 activity when compared to mTORC1. We detected different proliferative states between primary tumor and brain metastatic tumors by signal quantitation (Figure 6C). There was a higher number of proliferating cells in primary BC tumors, while the majority of cells in BC brain metastases were largely quiescent (Figure 6C).

### 2.5. mTORC2 Inhibition Affects BMRC Survival/Proliferation

**Figure 7:**
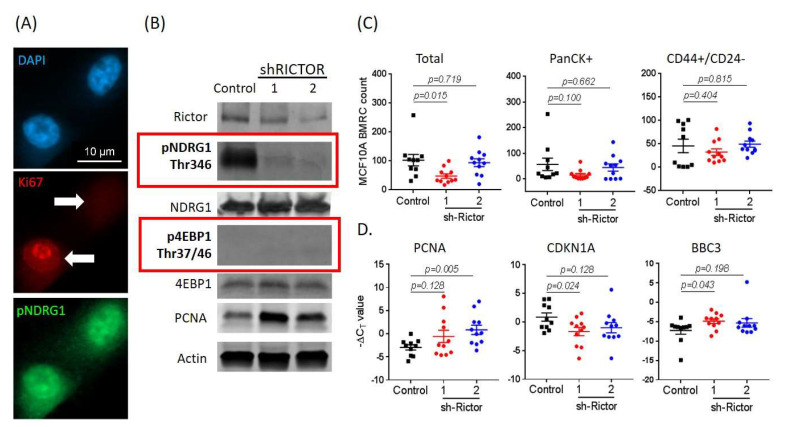
Inhibition of mTORC2 RICTOR decreases CTC proliferation markers. (**A**) High-definition immunofluorescence on MCF-10A cells showing differential expression of Ki67 proliferation marker, along with high pNDRG1 expression, indicative of active mTORC2 signaling (scale bar = 10μm). (**B**) Western blotting analyses of ShRNA RICTOR knockdowns of MCF10A cells showing that RICTOR knockdown resulted in decreased pNDRG1 expression, while p4EBP1 status remained unchanged (red boxes). Control denotes non-targeting scrambled control, and numbers 1 and 2 (below sh-RICTOR) denote two distinct lentiviral shRNA constructs against RICTOR. (**C**) (Top) Significant decrease of total BMRCs collected from ex vivo experiments using MCF-10A-shRICTOR knockdowns injected in NSG mice (1.0 × 10^5^ cells/mouse; *N* = 11). Conversely, no change of PanCK+ or CD44+/CD24− BMRC cell populations was detected. (**D**) (Bottom) Real-time PCR of ex vivo MCF-10A shRICTOR BMRCs exhibit increased gene expression for PCNA (proliferation marker), along with decreased CDKN1A (quiescence status) and increased BBC3 (PUMA) expression, consistent with a pro-apoptotic response (*N* = 11).

Next, to examine the effects of mTORC2 inhibition on BMRCs, we applied a genetic manipulation approach to MCF-10A breast cancer cells, a non-tumorigenic, ER+/PR+ cell line [43,44,45]. MCF-10A cells were stained with DAPI, Ki-67 and pNDRG1 (Figure 7A). Similar to patient-derived CTCs, these cells exhibited a variable proliferative status, defined by Ki-67^low^ vs. Ki-67^high^ staining (Figure 7A, middle panel). However, mTORC2 activity remained the same, as evidenced by pNDRG1+ staining (Figure 7A, bottom panel). Because RICTOR is an essential component of the mTORC2 complex [32,33,34,35,36], found to be activated in transcriptomic analyses, we employed shRNA to silence RICTOR expression, and studied effects of mTORC2 inhibition in MCF-10A cells. RICTOR knockdown attenuated mTORC2 activity, as evidenced by decreased pNDRG1 expression (Figure 7B). However, the use of shRICTOR did not affect mTORC1, as p4EBP1 status did not change. Lastly, we injected shRICTOR MCF10A cells in NSG mice. At 3 weeks post-injection, mice were sacrificed, visceral organs and bone marrow were collected, and cells were isolated using our FACS strategy (HLA-ABC, mammaglobin/CD15, and PanCK+ or CD44+/CD24₋ cell selection) (Figure 1). Staining of organs did not provide detectable evidence of metastatic colonization in control vs shRICTOR MCF10A-injected mice over a 3-months period (data not shown). Conversely, we found a significant decrease of total BMRC cells in animals injected with a shRICTOR MCF10A clone without any specific difference in epithelial vs stem-like BMRC populations (Figure 7C). Following RNA isolation, qPCR analysis for established mTORC2 targets showed a decrease in CDKN1A expression, and increased PCNA and BBC3 (PUMA) gene expression in shRICTOR MCF-10A cells (Figure 7D). Taken together, these data suggest that mTORC2 signaling is necessary for CTC implantation within the bone marrow and survival as CTC-derived BMRCs.

## 3. Discussion

This study provides first-time evidence identifying increased mTORC2 and decreased mTORC1 signaling in ex vivo BMRCs and CTCs, compared to de novo CTCs. Increased mTORC2 signaling could be a hallmark of human BMRCs. Second, analysis of BC CTC-derived xenografts (CDXs) showed that solitary BM and tissue-resident CTCs have high mTORC2 activity. Third, we show that augmented expression levels of mTORC2 downstream targets are found in quiescent CTCs (Ki67−/RBL2+ cells) of paired primary vs. brain metastatic BC tissues. Finally, shRNA knockdown of RICTOR, an essential component of mTORC2 signaling, increased CTC expression of Ki67 and PCNA, and established markers of proliferation, along with decreased with *CDKN1A* (marker of quiescence) expression. These results emphasize potential actions of RICTOR/mTORC2 on inhibiting proliferation and maintaining quiescence in CTCs/BMRCs. It will be interesting to explore whether these transcriptomic changes occur in tandem or reflect compensatory mechanisms to counteract genetic aberrations [46,47,48].

The work presented elucidates the molecular interplay driving dormancy or proliferation between breast cancer BMRCs and CTCs. Additionally, this work implicates the mTOR signaling pathway as a critical determinant promoting CTC seeding and maintaining long-term BMRC dormancy [2,3,49]. The time and clinical conditions of BC patients may affect how BMRCs undergo this transition and may account for the long latency periods and late relapses. While the specific mTORC1/mTORC2 molecular interplay is likely to be cancer type- and/or tissue type-dependent, rather than universal, our experimental strategies and findings can nevertheless provide an important framework to better understand the initial steps of the metastatic cascade, along with identifying markers and pathways responsible for the transition from cellular dormancy to a proliferative and potentially metastatic state [3,23,50].

Expression and functionality of the mTOR pathway were found to be associated with the proliferative switch of BMRC cells. Multiple oncogenic mutations are known to characterize benign breast cancers that do not metastasize [30,50]. Conversely, the activation of some oncogenic pathways repress BC metastasis, but increase tumor cell proliferation [3,50]. Our findings thus do not exclude the possibility that BC metastasis occurs from advanced, late stage tumors. We also cannot exclude other pathways, e.g., the Wnt pathway, which showed significant expression changes in our analyses and may be equally important (Table 2). Regardless, our findings are relevant and have particularly strong translational implications. They advance scientific understanding of metastatic mechanisms and roles of BMRC/CTC interactions, and can promote the use of specific mTOR inhibitors for novel therapies [51,52,53,54]. This study is not without limitations. First, analyses were performed on a limited number of patients; therefore, these observations cannot be generally applicable to breast cancer since not all BC subtypes may follow these models and pathways. Second, we profiled a limited number of BMRC/CTC populations. Although this was done stochastically, it may present sampling bias. Third, we did not perform mechanistic studies by injection of invasive-competent or metastasis-competent BC cells, e.g., MCF-7 or MDA-MB-231 cells, in NSG mice to monitor mTORC1/mTORC2 activities, and their effect on biomarkers of proliferation (Ki67/PCNA) and quiescence (RBL2/CDKN1A) in different phases of the metastatic cascade, and/or any association between mTORC1/mTORC2 signaling and genomic instability. Additional investigations are needed to clarify these aspects. However, our work can be viewed as a first step in deciphering CTC heterogeneity, focusing upon the distinction between dormancy-competent BMRCs and metastasis-competent CTCs, how the two intersect over time, and which regulatory mechanisms characterize these cell types. These investigations are also relevant in relation to shedding of CTCs from primary BC tumors, and their systematic spatiotemporal dissemination to BM and CTC seeding in distant organs. The overall CTC population in patient blood is likely derived from primary or metastatic tumors at different stages of evolution, along with the intermittent CTC dissemination from BM to other organs. Therefore, there is the need to characterize CTC subsets and CTC biomarkers further, e.g., investigating notions that Ki67 expression is a biomarker of CTC stage and/or CTC evolution. Because these distinct CTC subsets can all potentially possess metastatic capabilities, further molecular characterization of CTC subsets will be of paramount importance for the development of therapies targeting these seeds of metastasis.

## 4. Materials and Methods

### 4.1. Patient Blood Collection and Samples

Peripheral blood from patients diagnosed with mBC was provided according to protocols Pro00013429 and Pro00018256, approved by Institutional Ethical Review Board at Houston Methodist Research Institute (HMRI). A total of 20 patient samples were used in this study. Sixteen samples (2 mL-yielding 25-105 CTCs per sample) were used for flow analyses, 4 (16 mL) samples were used for in vivo injection into two mice each, and 2 samples were used for both flow analyses and in vivo selection. Thirty to thirty-five milliliters of peripheral blood was collected into EDTA or CellSave^®^ tubes under aseptic conditions according to the principles of the Declaration of Helsinki and after receiving a written consent from patients. Blood samples were provided immediately to the laboratory for CTC isolation and analysis. Paired archival primary and metastatic breast cancer tissues (pBC/mBC) were provided by the Cooperative Human Tissue Network, Southern Division (University of Alabama-Birmingham, Birmingham, AL, USA).

### 4.2. Cell Lines and Tissue Culture

Human breast cancer MCF10A and MDA231BR cell lines were freshly recovered from liquid nitrogen before they were used for indicated experiments (<6 months). The MCF10A cell line was procured from the Tissue Culture Core of Baylor College of Medicine (Houston, TX, USA). Brain metastasis-selected MDA-MB-231BR cell variant was kindly provided from Dr. Patricia Steeg’s laboratory (National Institutes of Health, Bethesda, MD, USA). The 231BR clone is the result of sequential cycles of injection of MDA-MB-231 parental cells in nude mice with increased propensities to form brain metastasis in these animals [44]. Cells were cultured in Dulbecco’s modified Eagle’s medium plus F12 (DMEM/F12; Invitrogen, Carlsbad, CA, USA) supplemented with 10% FBS (Invitrogen). MCF-10A cells (obtained from the Cell Core facility of Baylor College of Medicine, Houston, TX) were cultured in mammary epithelial basal medium (MEBM) containing 10% FBS and 10 ng/mL of human EGF, 5 mg/mL of insulin, and 1 mg/mL of hydrocortisone. All cells were cultured in a humidified 5% CO_2_ incubator at 37 °C, tested every month for *mycoplasma* contamination and used only at low passage and if *mycoplasma* negative. All cell lines were regularly examined by microscopy for phenotypic changes before their use.

### 4.3. Flow Cytometry

Peripheral blood mononuclear cells (PBMCs) were isolated from either patient (8 mL), CDX (500–800 µL) blood, or animal BM (500–800 µL) using red blood cell (RBC) lysis buffer (154 mmol/L NH_4_Cl, 10 mmol/L KHCO_3_, 0.1 mmol/L EDTA). PBMCs were stained with marker antibodies, then analyzed using FACSAriaII flow cytometer (BD Biosciences, San Jose, CA, USA) for multi-parametric selection [21]. Briefly, forward scatter area vs forward scatter height was used for doublet discrimination. DAPI staining was used to determine cell viability. We used conjugated antibodies FITC-CD45 (#304054; 1:200 dilution), FITC-CD34 (#343504; 1:200 dilution), FITC-CD105 (#323204; 1:200 dilution), FITC-CD90 (#328108; 1:200 dilution), and FITC-CD73 (#344016; 1:200 dilution) obtained from BioLegend (San Diego, CA, USA). FITC positive cells were eliminated from the downstream analyses. For ex vivo cell isolation, conjugated antibodies APC-Cy7-CD44 (#103028, 1:100 dilution), BV650-CD44 (#103049, 1:100 dilution), AF647-Pan-cytokeratin (#628604, 1:100 dilution), and FITC HLA-A/B/C (#311404; 1:200 dilution) from Biolegend were used. The PE-Pan-cytokeratin (#5075, 1:100 dilution) antibody was from Cell Signaling Technology, Danvers, MA, USA. The dilution for each antibody listed above was empirically determined using appropriate negative and positive controls. The compensation matrices were generated by using unstained and single fluorophore-stained Versacomp beads (Beckman Coulter Life Sciences, Indianapolis, IN, USA), and applied prior to sorting. FACS data were analyzed by FlowJo_Version 10 (Ashland, OR, USA).

### 4.4. CellSearch Analyses and CTC Enumeration

Peripheral blood (7.5 mL) was collected into CellSave^®^ tubes and EpCAM-positive CTCs were captured and enumerated by the FDA-cleared CellSearch^TM^ platform (Menarini Silicon Biosystems Inc., Huntingdon Valley, PA, USA) following the manufacturer guidelines, according to the CTC definition by CellSearch (EpCAM+/CK+/DAPI+/CD45− cells). CTC enumeration of blood from NSG mice was performed using 7.0 mL of blood from healthy donors spiked with 500 µL of mouse blood using the CellSearch Circulating Epithelial Cell Kit (Menarini Silicon Biosystems, Inc.). Fluorescent CTCs were imaged and enumerated by the automated CellBrowser software (Menarini Silicon Biosystems, Inc.) according to the manufacturer guidelines.

### 4.5. CTC-Derived Xenografts

The generation of CTC-derived xenografts (CDXs) was performed using 4–8-weeks-old NOD.Cg-Prkdcscid Il2rgtm1Wjl/SzJ (NSG) immunodeficient mice (Jackson Labs, Bar Harbor, ME, USA) according to the Institutional Animal Care and Use Committee (IACUC) protocol IS00004851 approved by HMRI. Flow-sorted lineage-negative PBMCs (~5.0 × 10^4^ cells) from 8.0 mL of patient blood were administered via intracardiac injection into anesthetized NSG mice under aseptic conditions. Previous studies demonstrated that immunodeficient mice injected with BC cell lines (0.5–1.0 × 10^6^ cells; 1.0 × 10^5^ MCF10A cells were injected per NSG mouse in our experiments) with different metastatic abilities normally develop overt macro-metastases in 3–6 weeks. Considering that CTCs are rare, mice were sacrificed 4–8 months after CTC injection (vs 3 months following MCF10A cell injections) with patient-derived, FACS-enriched CTC populations. This timeframe was considered sufficient for in vivo selection, including the elimination of non-malignant cells, and the successful formation of resident single cells and micrometastasis in BM or other organs. Mice were anesthetized, then sacrificed, and approximately 800–900 µL of peripheral blood was collected into EDTA tubes. The femur and tibia were isolated, and BM was obtained by flushing the femur and tibia with 1×PBS containing 5 mM of EDTA using 28G×½ needles, followed by centrifugation at 300 × *g* for 10 min. PBMCs were isolated immediately from the blood and BM for FACS analyses, as previously reported [28,29]. Different organ tissues were also harvested and fixed in 4% paraformaldehyde for downstream analyses.

### 4.6. RNA Microarrays and Pathways Analysis

Flow-sorted BMRC and CTC populations were processed for RNA isolation using NucleoSpin^®^ RNA isolation kit (Macherey-Nagel Inc., Bethlehem, PA, USA). RNA quality was verified by the RNA integrity index (28S/18S ribosomal peaks and their ratio) at MD Anderson Cancer Center Sequencing and Non-coding RNA Core facility (Houston, TX, USA). Microarray hybridization was performed using the Human Transcriptome Array 2.0 (HTA_2.0) platform (Affymetrix, Santa Clara, CA, USA). Files were normalized and analyzed by Transcriptome Analysis Console, version 4.0.1 (Affymetrix). Microarray data is available from the Gene Expression Omnibus as GSE150624. https://www.ncbi.nlm.nih.gov/geo. Pathway enrichment analyses for significant pathways and upstream regulator were performed by Ingenuity Pathway Analysis, version 0.7 (Qiagen, Germantown, MD, USA), as previously reported [21].

### 4.7. Immunofluorescence, Immunohistochemistry and DEPArray

FACS-sorted BMRCs/CTCs were fixed with 4% paraformaldehyde, permeabilized with 0.05% Triton X-100 and stained for immunofluorescence (IF) using selected primary and secondary antibodies, according to published procedures [21]. Alexafluor (AF) 594 (red) and AF-488 (green) tagged secondary antibodies against mouse and rabbit primary antibodies were used for IF. Bright-field and fluorescent microscopic images were captured using Zeiss Axio Observer microscope Z1 (Carl Zeiss, Jena, Germany) and data were analyzed by ZEN2 software (Carl Zeiss). For DEPArray, FACS-isolated cells were subsequently washed with SB115 buffer, loaded into the DEPArray cartridge, and imaged with 10× objective (Menarini Silicon Biosystems, Inc.). Data were analyzed using the custom Fixed_Low_Density program of the DEPArray v3.0 platform. CTCs were visualized by DAPI nuclear staining, Ki67, and other markers as listed. DEPArray positive/negative IF values were determined by CellBrowser^TM^ software, which calculate multiple parameters (background, mean fluorescence, etc.) during cell detection. Intensity threshold settings are then automatically applied to captured images, equalizing fluorescence levels in all images, but without altering primary fluorescence intensity values. Proliferative status of CTCs was evaluated by mean fluorescence intensity using FlowJo ver 10 software. Imaging parameters were established using control cells, and all subsequent images were captured using the same settings.

For immunohistochemistry (IHC), harvested tissue was fixed and stained. Sources of antibodies were: HLA-ABC (#565292) and mouse anti-cleaved PARP (Asp214) (#552596) were received from BD Biosciences San Jose, CA, USA; GCDFP-15 mammaglobin cocktail (#906H-08) from Sigma, St. Louis, MO, USA; Rabbit anti-RBL2 (#ab76234) from Abcam, Cambridge, MA, USA; Rat anti-Ki67 (#TA801577) from Origene, Rockville, MD, USA; Rabbit anti-pNDRG1 (#5482) from Cell Signaling Technology; and mouse anti-p70s6K1 (#MABS82) from EMD Millipore, Burlington, MA, USA; Anti-mitochondrial antibody (#MSM1-739-P), CD44 (#960-MSM2-P), PanCK (#MSM2-371-P), and Muc1 (#4582-MSM18-P) antibodies from Neobiotechnologies, Union City, CA, USA; and CD298 (#GTX114272) from Genetex, Irvine, CA, USA. All histochemical and antibody staining was performed by the HMRI Research Pathology Core. IHC on mouse tissue using antibodies of mouse origin were performed using M.O.M. elite peroxidase kit; dual IHCs were performed using ImmPRESS Duet Double Staining HRP/AP Polymer Kit, and triple IHCs were performed by multiplexing with ImmPRESS-AP Anti-Rat IgG, Mouse Adsorbed Polymer Detection Kit (Vector Labs, Burlingame, CA, USA). Slides were imaged using an EVOS XL Cell Imaging System (ThermoFisher Scientific) and quantified using ImageJ software by the Pathology Core facility at Houston Methodist Hospital (Houston, TX, USA) [19,20,21,22].

### 4.8. ShRNA, qPCR, and Western Blotting Analyses

Lentiviral pLKO.1 shRICTOR (plasmid #1853 and #1854) and scrambled shRNA (plasmid #1864) constructs were obtained from AddGene (Cambridge, MA, USA), packaged in HEK293T cells using 3^rd^ generation lentiviral packaging system, and transduced into MCF10A cells [41]. Cells were treated with puromycin (5 μg/mL) for 7 days, and used for subsequent experiments.

Total mRNA and cDNA amplification were carried out from isolated RNA using REPLI-g single-cell WTA amplification kit, according to manufacturer protocols (Qiagen, Inc.). Amplified cDNA was purified employing the ExoSAP method (Affymetrix; #78202.4X.1.ML), and subjected to qPCR using the SensiFAST™ SYBR^®^ Hi-ROX Kit (Bioline, #BIO-92020). CT values were normalized with housekeeping gene beta-actin and plotted as -ΔC_T_ values. A paired Student’s *t*-test was performed to obtain the *p*-value of each gene. Primer sequences for each gene were obtained from the PrimerBank and shown in Table S1 [55].

For Western blotting analyses, 20 µg of proteins were resolved by 4–15% gradient SDS-PAGE and transferred to polyvinylidene difluoride membranes (Bio-Rad, Hercules, CA, USA). Blots were then blocked with LI-COR blocking buffer, incubated with primary antibodies, followed by IR-dye conjugated secondary antibodies, and images captured using LI-COR Odyssey (LI-COR biotechnology, Lincoln, NE, USA). Primary antibodies for Western blots: Rictor (#2114), NDRG1 (#9485), phospho-NDRG1 (#5482) 4EBP1 (#9644), phospho-4EBP1 (#2855), and PCNA (#2586) were obtained from Cell Signaling Technology. Anti-beta actin (#sc-69879) antibody was obtained from Santa Cruz Biotechnology. Greyscale images of original Western blots membranes are available in Figure S3.

## 5. Conclusions

In summary, we have used patient-derived CTCs to re-create the landscape of metastatic dormancy in immuno-compromised mice. Using this model, we have been able to identify, isolate, and characterize solitary breast cancer cells in bone marrow (BMRCs) and target organs. This allowed for whole genome transcriptomic arrays to identify mTORC2 signaling a prime candidate for maintaining dormancy in metastatic breast cancer cells. These findings are relevant because: (1) there is no model of metastatic dormancy that can faithfully recount the complete spectrum of heterogeneous CTCs, proliferative tumorigenic cells, as well as dormant clones present in metastatic breast cancer patients; (2) studies based on using cell lines may not capture the intricacies of prolonged dormancy due to accumulated genetic aberrations; and (3) complexities of CTC signaling pathways responsible for metastatic initiation may be obscured and/or overlooked when utilizing macro-metastasis onset as an end point. We have established proof-of-concept investigations to determine initial CTC colonization capacities, their ability to survive in a foreign microenvironment such as bone marrow, and their tumorigenic potencies in vivo. Further characterization of these solitary cells and cell populations will potentially enable the identification and isolation of quiescent CTCs directly from patient blood, monitoring of asymptomatic progression during tumor metastasis, and promoting early interventional therapies.

## Figures and Tables

**Table 1:** Number and biomarker definition of ex vivo BMRCs and CTCs.

mBC Pt #	Tumor Subtype	Length of in vivo Selection (days)	BMRCs				CTCs			
			CD44+/CD24−	PanCK+	CD44+/CD24− and PanCK+	Total	CD44+/CD24−	PanCK+	CD44+/CD24− and PanCK+	Total
4	ER+/PR+	264	60	101	583	744	7	1272	138	1417
17	ER+/PR+	160	40	323	97	460	38	240	22	300
18	HER2+	72	3	111	106	220	10	39	1	50
19	HER2+	140	40	96	53	189	40	417	31	488
8	TNBC	97	0	50	176	226	163	48	14	225
20	TNBC	64	64	96	478	638	7	1345	86	1438

**Table 2:** Top activated and inhibited canonical pathways and cellular functions in ex vivo cells.

Canonical Pathways	−log (*p*-value)	z-score	Functions Annotation	*p*-value	z-score
mTOR Signaling	8.06	0.632	cancer cell death	1.59 × 10^−10^	2.771
Wnt/β-catenin Signaling	2.91	1.897	osteosarcoma cell death	4.08 × 10^−9^	4.082
Cardiac β-adrenergic Signaling	2.28	0.905	tumor necrosis	1.17 × 10^−8^	3.001
EIF2 Signaling	16.4	−4.025	metastatic solid tumor	1.37 × 10^−8^	−2.567
CD28 Signaling in T Helper Cells	4.25	−1.265	cell invasion	1.92 × 10^−8^	−2.448
Cdc42 Signaling	3.14	−0.816	tumor cell line invasion	5.24 × 10^−8^	−2.368

IPA performed with gene log fold changes −2<>2, ANOVA *p*-value < 0.05.

**Table 3:** Upstream regulators of ex vivo cells.

Regulator	Molecule Type	z-score	*p*-value of Overlap
Rapamycin	chemical drug	4.849	8.74 × 10^−25^
5-fluorouracil	chemical drug	3.64	8.06 × 10^−18^
CD 437	chemical drug	5.34	3.82 × 10^−17^
ST1926	chemical drug	4.904	1.4 × 10^−14^
RICTOR	other	4.842	1.61 × 10^−13^
MYCN	transcription factor	−3.69	5.81 × 10^−15^
LPS	chemical drug	−3.058	8.38 × 10^−13^
IFNG	cytokine	−3.229	1.01 × 10^−7^
poly rI:rC-RNA	biologic drug	−3.535	2.17 × 10^−7^
TCR	complex	−2.331	2.81 × 10^−7^

IPA performed with gene log fold changes −2<>2, ANOVA *p*-value < 0.05.

## Supplementary Materials

The following are available online at https://www.mdpi.com/2072-6694/12/6/1626/s1, Figure S1: (A) Ki67 and (B) uPAR and int-β1 expression in epithelial (PanCK+) and stem-like (CD44+/CD24−) breast cancer CTCs, Figure S2: Median fluorescence intensity of integrin-β1 in epithelial (PanCK+) and stem-like (CD44+/CD24−) CTCs, Figure S3: Greyscale images of original western blot membranes with molecular weight markers, Table S1: List of primers used for qPCR analysis.
