# Supplementary material for: Molecular Interplay between Dormant Bone Marrow-Resident Cells (BMRCs) and CTCs in Breast Cancer
Source: Cancers (Basel). 2020 Jun 19;12(6):1626. doi: 10.3390/cancers12061626 (PMC7352937; doi:10.3390/cancers12061626)
Supplement: Supplementary file 1 [file cancers-12-01626-s001.pdf]

SupplementaryMaterials:

Molecular Interplay between Dormant Bone Marrow-Resident Cells (BMRCs) and CTCs in Breast Cancer

Debasish Boral, Haowen N. Liu, S. Ray Kenney and Dario Marchetti

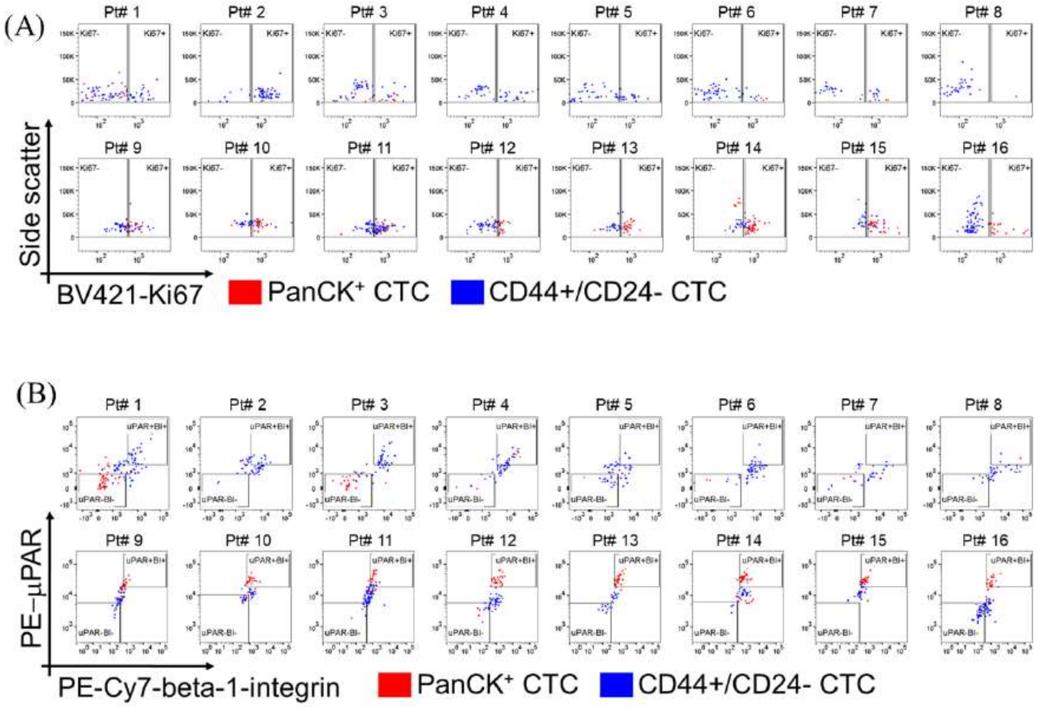

Figure S1. (**A**) Ki67 and (**B**) uPAR and int-β1 expression in epithelial (PanCK+) and stem-like (CD44+/CD24-) breast cancer CTCs. Dot plots from all 16 patients.

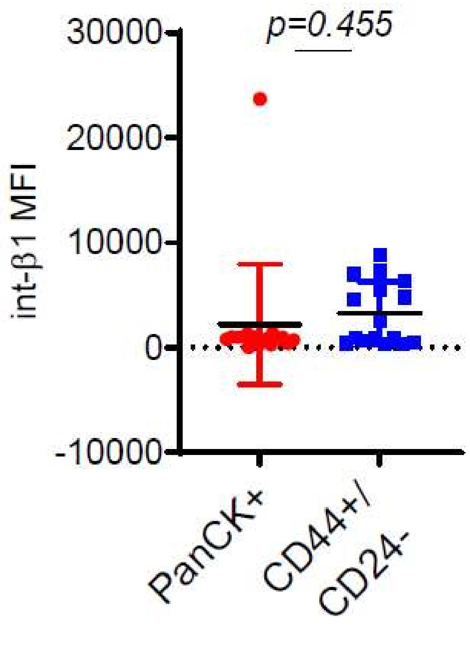

Figure S2. Median fluorescence intensity of integrin-β1 in epithelial (PanCK+) and stem-like (CD44+/CD24-) CTCs.

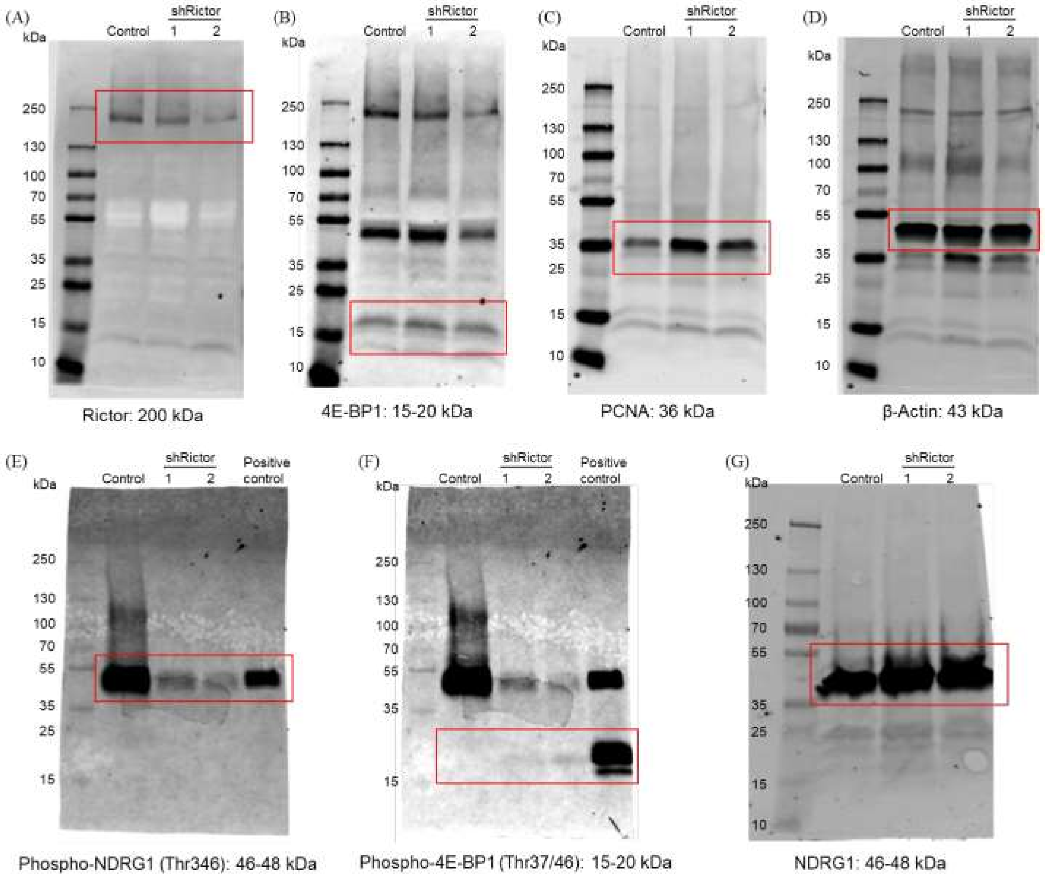

Figure S3. Greyscale images of original western blot membranes with molecular weight markers. Membranes (**A**) and (**B**) are the same membrane first probed with Rictor, and later with the 4EBP1 antibody. Membranes (**C**) and (**D**) are the same membrane first probed with PCNA, and later with the β-Actin antibody. Membranes (**E**) and (**F**) are the same membrane probed first with phospho-NDRG1 and later with phospho-4EBP1. Membrane (**G**) probed with NDRG1 antibody. Cell lysates from MDA-MB-231 cells treated with 100nM recombinant human insulin for 30 minutes was used as a positive control for membranes (**E**) and (**F**).

Table S1. List of primers used for qPCR analysis.

Gene	GenBank Accession	Amplicon size	Forward primer	Reverse primer	PrimerBank ID
BBC3	NM_014417	98	GACCTCAACGCACA GTACGAG	AGGAGTCCCATGATGA GATTGT	15193488a1
CDC42	NM_044472	128	CCATCGGAATATGTA CCGACTG	CTCAGCGGTCGTAATCT GTCA	89903014c1
CDKN1A	NM_000389	139	TGTCCGTCAGAACCC ATGC	AAAGTCGAAGTTCCATC GCTC	310832423c1
EIF4B	NM_001417	140	GGCTGATGAAACGG ATGACCT	GGTCGATATTGGGTTCC CGA	148746207c2
PCNA	NM_182649	109	CCTGCTGGGATATTA GCTCCA	CAGCGGTAGGTGTCGAA GC	33239449b1
GAPDH	NM_002046	102	AAGGTGAAGGTCGG AGTCAAC	GGGGTCATTGATGGCAA CAATA	83641890b1
